# Guidelines for the successful generation of protein–ligand complex crystals

**DOI:** 10.1107/S2059798316020271

**Published:** 2017-02-01

**Authors:** Ilka Müller

**Affiliations:** a Structural Biology, Discovery from Charles River, Chesterford Research Park, Saffron Walden CB10 1XL, England

**Keywords:** protein–ligand complexes, protein crystallization, construct design, soaking

## Abstract

With continuous technical improvements at synchrotron facilities, data-collection rates have increased dramatically. This makes it possible to collect diffraction data for hundreds of protein–ligand complexes within a day, provided that a suitable crystal system is at hand. However, developing a suitable crystal system can prove challenging, exceeding the timescale of data collection by several orders of magnitude. Firstly, a useful crystallization construct of the protein of interest needs to be chosen and its expression and purification optimized, before screening for suitable crystallization and soaking conditions can start. This article reviews recent publications analysing large data sets of crystallization trials, with the aim of identifying factors that do or do not make a *good* crystallization construct, and gives guidance in the design of an expression construct. It provides an overview of common protein-expression systems, addresses how ligand binding can be both help and hindrance for protein purification, and describes ligand co-crystallization and soaking, with an emphasis on troubleshooting.

## Abstract 2

This article aims to guide efforts in protein–ligand complex crystal structure generation, with special consideration of protein construct design, and summarizes different approaches to co-crystallization and crystal soaking. Common problems and pitfalls are highlighted.

## 1. Introduction   

Over the last years, experimental information on the vast number of protein-crystallization experiments carried out by different structural genomics initiatives (Savitsky *et al.*, 2010 ▸; Ng *et al.*, 2016 ▸) and also within industrial settings (Öster *et al.*, 2015 ▸) has become publically available. Systematic analysis of this large knowledge base has helped to generate a catalogue of strategies for the crystallization of proteins, especially as the data include negative results, *i.e.* which approaches did not lead to the desired outcome. In addition, improvements to the molecular-cloning toolbox and automation as well as miniaturization of the crystallization setup make it possible to parallelize experiments, thus improving the timelines from choosing the target to solving the first crystal structure. This is particularly important if the structure is not the end point, but rather the start for the generation of multiple or even hundreds of ligand-bound structures to guide drug discovery.

About 75% of the >100 000 protein crystal structures in the Protein Data Bank contain at least one of nearly 20 000 unique ligands, some of them unintentionally as a result of the purification or crystallization process, and others added deliberately to study protein function or as part of structure-based drug design (http://www.wwpdb.org; Berman *et al.*, 2000 ▸). This large proportion reflects the fact that ligands are usually well tolerated in crystal structures. The ligands might even be required for crystal formation, or bind to the protein during protein expression and purification, from where they are carried through into the crystals. Yet, just as there is not one single way to successfully crystallize a protein, there is not one way to generate the structure of a particular protein–ligand complex.

This paper aims to give an overview of the key considerations for protein–ligand complex crystal formation. It is enriched with examples from both the literature as well as cases in which members of the structural biology team at Charles River were involved.

In the proceedings of the 2006 CCP4 Study Weekend, Hassell *et al.* (2007 ▸) detail how they approach the problem of protein–ligand complex crystal growth in relation to the question ‘when to add ligand to the protein?’. The authors give excellent guidance on co-expression, co-purification, co-crystallization and soaking of ligands, and their suggestions are still very relevant to date. This article is going to take a step back, and starts with the question ‘which protein construct to use?’. Particular emphasis will be given to protein construct design and protein engineering in order to overcome some of the issues hindering complex crystal formation. It will evaluate how the presence of ligands during protein expression or purification can be both beneficial and obstructive, and factors that need to be considered during co-crystallization and ligand soaking will be discussed.

Even when a ligand complex structure has already been determined for the target protein, it might not be straightforward to generate one with another ligand. Often, inspection of the structure can reveal warning signs of possible caveats with a particular protein construct or crystal system, and examples will be discussed throughout the paper.

## 2. Overview   

**Figure 1:**
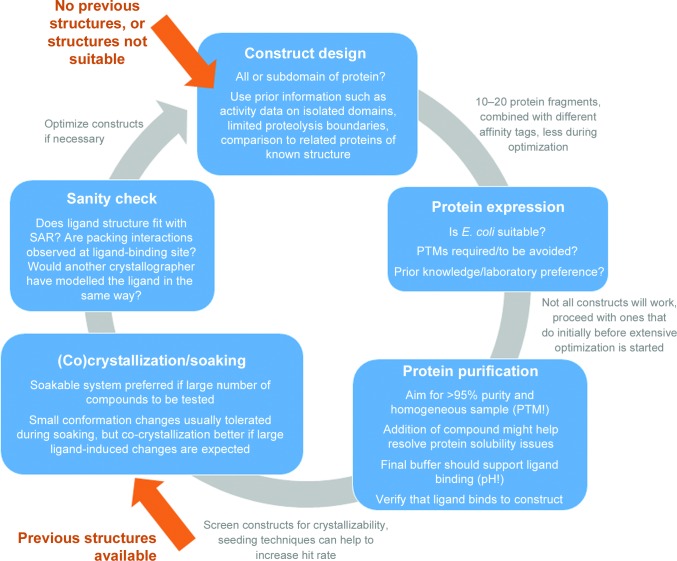
Experimental cycle for protein–ligand complex crystal structure generation. For targets with limited structural information, the cycle starts with selecting suitable start and end points for the protein (subdomain) of interest. The resulting protein fragments can be combined with different expression vectors, adding affinity tags for purification. Not all of these constructs will express equally well, and usually only the subset with sufficient expression levels will be taken forward into purification. Extensive optimization of expression and purification conditions should be weighed against the design of more constructs and the use of different solubilizing and affinity tags. If previous structures of the protein (fragment) are available, the cycle is typically entered at the crystallization or soaking stage. Co-crystallization and soaking is ligand-dependent, even for ligands of similar binding affinity. If available, testing batches of 3–5 similar compounds in parallel is recommended. After a co-crystal structure has been determined, the ligand complex should be carefully checked and, if necessary, the cycle re-entered.

Generation of protein–ligand complex crystals can be divided into five main steps: construct design, protein expression and purification, protein–ligand complex crystal formation and finally a sanity check of the complex structure. It is important to use as much prior information on the protein target as possible alongside general principles, and to keep in mind that not all factors leading to protein crystals are understood. Thus, some problems may only be solved by trial and error, and the design cycle shown in Fig. 1 ▸ is repeated several times. With prior structural information available, it might be possible to skip these first steps and enter the cycle at the complex crystal formation step. Experimental considerations for the different entry points are given in Table 1 ▸.

## 3. Construct design and protein engineering   

To study protein–ligand inter­actions, it is often not necessary to investigate the ligand in the context of the full-length protein, especially if it is comprised of multiple domains (Derewenda, 2010 ▸). In the case of the somatic mutation underlying chronic myelogenous leukaemia (CML), parts of the c-Abl gene are fused to the breakpoint cluster region (Bcr), resulting in the approximately 200 kDa BCR-Abl oncogene. This fusion protein is a constitutively active form of the tightly regulated tyrosine kinase c-Abl (1130 amino-acid residues), which results in the uncontrolled cell growth and survival in leukaemia. The structure of the kinase domain of Abl (less than 300 residues) was used by Schindler *et al.* (2000 ▸) to elucidate the structural mechanism of BCR-Abl inhibition by STI-571 (Gleevec^TM^/imatinib), and it was found that the drug binds to and stabilizes the inactive form of the kinase (PDB entry 1fpu). Using Abl kinase-domain constructs further helped to understand imatinib-resistant mutants in CML patients and assisted the development of inhibitors that overcome this drug resistance (Levinson *et al.*, 2006 ▸; Cowan-Jacob *et al.*, 2007 ▸).

**Figure 2:**
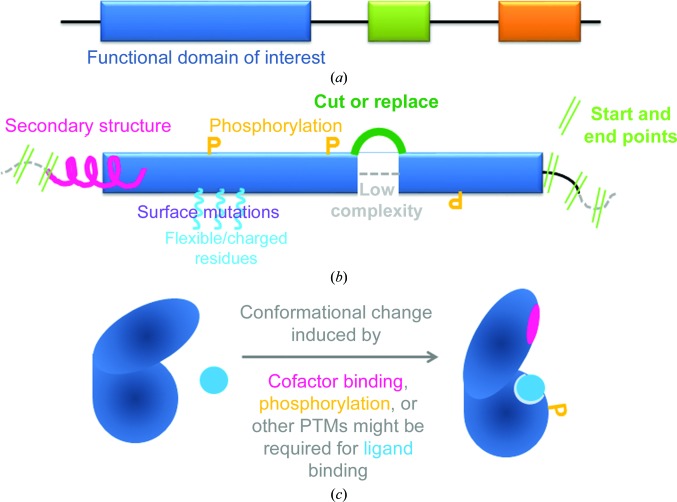
Cartoon representation of the construct-design process. (*a*) For multi-domain proteins, the domain of interest is identified using experimental and/or alignment data. (*b*) The domain architecture of the isolated domain is then inspected for suitable start and end points (bright green), avoiding cutting through secondary-structure elements (magenta) and with the aim of including all residues required for function. Limited proteolysis data as well as secondary-structure prediction tools can be used as a guide, as well as structural data of homologue proteins. Sample homogeneity can be achieved at the sequence level through the mutation of residues targeted by post-translational modifications (PTMs) such as phosphorylation (orange) that either prevent or mimic the PTM. Further construct optimization can involve the mutation of surface residues with flexible or charged side chains (cyan), either as single mutants or in clusters, to alanine or residue types that reverse or remove the charge. Regions of low structural complexity (grey, dark green) can be replaced by short linker residues or equivalent residues in homologue proteins to reduce conformational variability in the construct. (*c*) Events such as cofactor binding or PTMs can affect the conformational state and ligand-binding ability of the protein and should be considered in the design of the experiment.

In addition to identifying the ligand-binding domain, features relevant to crystallization need to be considered (Fig. 2 ▸). In our experience, testing many different protein constructs in a few initial 96-well crystallization screens is generally more successful in identifying crystal hits than screening only one construct under thousands of crystallization conditions. Starting with multiple constructs that potentially lead to several crystallization hits also helps in switching to a different crystal system if issues such as packing artefacts transpire. The number of constructs to be tested depends on the capacity of the individual laboratory for parallelization, and it should be kept in mind that not all of the protein constructs will lead to successful protein production. A systematic analysis published by the SGC in Oxford (Savitsky *et al.*, 2010 ▸) shows that less than half of the protein constructs entering expression trials yielded protein samples suitable for crystallization studies, and the authors suggest the design of 10–20 different protein constructs. Data mining by the SGC in Stockholm (Sagemark *et al.*, 2010 ▸) found that within their group an average of 11 constructs were designed per novel structure. For novel target proteins, we try at least two (but prefer three or four) different start and end points in combination with 2–3 different expression vectors, adding an N-terminal or C-terminal histidine tag.

To help prioritize different aspects of construct design, this chapter is divided into five subsections. Focusing on §§3.1 and 3.2, defining protein boundaries and taking conformational states into account, will help to keep the number of initial constructs in a range feasible for a laboratory with minimal automation or prior experience. §§3.3 and 3.4, on regions of disorder and surface mutations, might be worth considering if some of the initial protein constructs can be expressed and purified but are recalcitrant to crystallization. §3.5 is aimed at helping to make changes to the original construct when its crystal structure is rendered inappropriate for the aspect in question.

In this context, Savitsky *et al.* (2010 ▸) suggested the termin­ology ‘domain’ when addressing the PFAM-annotated structural domain and ‘protein fragment’ when discussing the boundaries within the sequence of the target protein. ‘Protein construct’ refers to the protein expressed, which might have additional residues compared with the protein fragment, depending on the choice of the expression vector.

The protein construct might carry an affinity tag to aid in protein purification, but the tag can also help to increase expression levels and solubility. The most commonly used tag is either a (cleavable) N-terminal or C-terminal hexahistidine (His_6_) tag. For the cleavable His_6_ tag, we usually try crystallization with and without the His_6_ tag removed. Using a cleavable GST (glutathione *S*-transferase) or MBP (maltose-binding protein) tag has the advantage that affinity purification generally results in higher purity of the sample compared with the His tag, and addition of an extra domain can further help with protein-expression levels and solubility. Hammarström *et al.* (2002 ▸) compared the effect of seven N-terminal expression tags on the expression levels and solubility of 32 human proteins of unknown structure. None of the tags stood out, but the larger tags generally yielded higher expression levels and solubility than an N-terminal His_6_ tag.

We typically remove these larger tags before crystallization; however, they have been found to aid in crystal formation. In these cases, the correct choice of (a short) linker was often crucial (Clifton *et al.*, 2015 ▸).

There are other affinity tags available, for example FLAG or streptavidin tags, but largely owing to the higher costs of resin or eluting agent they are more typically used as alternative backup systems (Savitsky *et al.*, 2010 ▸; Lichty *et al.*, 2005 ▸).

### 3.1. Start and end points   

The first step in construct design is to specify which domain(s) is (are) necessary for ligand binding or which function of the protein is being targeted by the ligand, as discussed above for BRC-Abl. The aim from a crystallo­graphic perspective is to identify the boundaries of the structured domain necessary to achieve ligand binding or maintain protein function, while excluding large unstructured regions which might hinder crystal formation. A good starting point is sequence alignment with closely related proteins of known structure, or the use of domain-recognition tools such as *PFAM* (Finn *et al.*, 2014 ▸) or *pDomTHREADER* (Lewis *et al.*, 2013 ▸).

In addition, secondary-structure and disorder prediction tools such as *PSIPRED*, *RONN* or *DISOPRED* (Buchan *et al.*, 2013 ▸; Yang *et al.*, 2005 ▸) can be used to avoid ‘cutting’ into continuous secondary-structure elements and to minimize the incorporation of long disordered regions at the boundaries. The amino-acid composition at the termini of the protein fragment is also worth considering with respect to the occurrence of serine, proline or aromatic residues. Savitsky *et al.* (2010 ▸) analysed the amino-acid composition at the termini of different protein fragments compared with the termini of the corresponding full-length proteins. They found an over-representation of proline and serine residues, and an under-representation of hydrophobic residues, in the protein fragments which successfully crystallized. These residue types are also reported to have higher than average propensity for disorder (Linding *et al.*, 2003 ▸). It can be speculated that the higher flexibility at the very termini might possibly contribute to construct solubility and fewer conformational restraints during crystal packing.

Once initial protein fragments have been designed, algorithms aimed at scoring these in terms of their probability to crystallize can be incorporated. For example, the data generated by structural genomics efforts on all of the protein constructs tested *versus* successful constructs were used to inform machine-learning approaches as implemented in *XtalPred-RF* (Jahandideh *et al.*, 2014 ▸) for scoring different protein constructs.

One should keep in mind that the methods used for these predictions bear some degree of uncertainty, and the design and testing of multiple constructs around the predicted boundaries are recommended.

### 3.2. Conformational state   

Many proteins depend on a chemical modification or a binding partner to switch between active and inactive states; the transition is often associated with extensive conformational change.

For protein kinases, the largest protein family in eukaryotes (Nolen *et al.*, 2004 ▸), a number of different types of inhibitors have been developed. Type 1 (ATP-competitive) inhibitors bind to the kinase ATP-binding site and inhibit the protein in its active conformation. Non-ATP-competitive inhibitors inhibit the enzyme by stabilizing an inactive conformation through binding to a pocket that is not present in the active state. For some kinases, the switch between an inactive and the active conformation requires phosphorylation in the so-called activation loop, a flexible polypeptide region in the vicinity of the ATP and protein substrate-binding site, while others can also adopt the active state if nonphosphorylated (Cowan-Jacob *et al.*, 2007 ▸). This means that for kinases, but also many other proteins, a further variable in the ligand co-crystallization experiment is the phosphorylation state, which will depend on the protein expression platform. It is not uncommon to find a mix of different phosphoryl­ation states in a protein prep­aration, and also variation in the phosphoryl­ation patterns between different protein preparations. As the resulting sample heterogeneity can hinder crystallization, it is recommended to separate the different states by ion-exchange chromatography. To shift the ratio towards the desired phosphorylation state, the protein can either be co-expressed with a phosphatase, phosphorylated *in vitro* or dephosphorylated during the purification process. Also, the presence of inhibitor during protein expression can have an effect on the phosphorylation profile. Cowan-Jacob *et al.* (2007 ▸) showed that Abl kinase is heterogeneously phosphorylated when expressed in Sf9 insect cells. Upon the addition of a kinase inhibitor during expression, they found that the amount of non­phosphorylated protein is increased, and in some cases also the overall protein yield. Alternatively, mutations can be introduced that either prevent phosphorylation by removing the site of phosphoryl­ation or mimic it by introducing a negative charge (Mace *et al.*, 2013 ▸).

Conformational changes can also be induced through co-activators or repressors, and this might need to be considered in structural studies of ligand complexes. In the case of HCV NS3 protease, the activity of NS3 increases upon binding of the HCV NS4 peptide by anchoring the active-site residues, and crystal structures of NS3 bound to different protease inhibitors have been generated using the NS4 peptide, either by addition of the NS4 peptide to the NS3 protein prior to crystallization (Yan *et al.*, 1998 ▸) or by constructing an NS3/NS4 fusion protein (Romano *et al.*, 2012 ▸).

### 3.3. Regions of disorder   

Within protein domains, there can be long stretches of disordered regions, which may hinder crystallization. In these cases, more sophisticated protein engineering might become necessary to displace these disordered residues. One approach would be to replace these regions of low complexity with equivalent residues of homologue proteins that are known or predicted to be less flexible.

**Figure 3:**
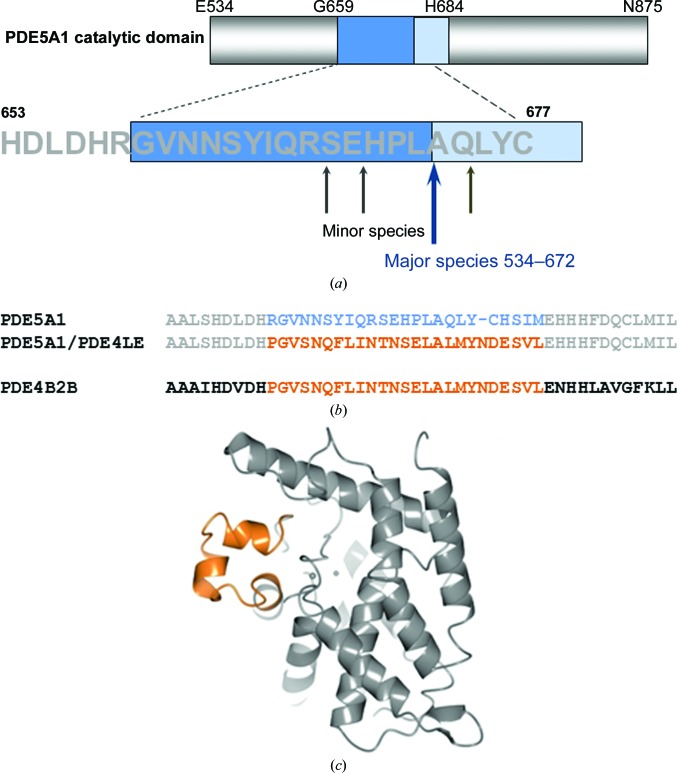
Construct design of the PDE5/PDE4 chimera protein. (*a*) Domain boundaries of the original PDE5A1 catalytic domain construct with disordered residues highlighted in blue and the proteolytic sites marked. (*b*) Sequence alignment between the loop region of PDE5A1 (top row) with equivalent residues in PDE4B2B (bottom row) and the sequence of the PDE5A1/PDE4B2B chimera protein (middle row). (*c*) Cartoon representation of the crystal structure of PDE4B2B (PDB entry 4nw7) with the loop insertion region highlighted in orange, created using *CCP*4*mg* molecular-graphics software (McNicholas *et al.*, 2011 ▸).

An example of the impact of flexible regions is the human cGMP-specific phosphodiesterase PDE5A1, which is the target of the drug sildenafil (Viagra^TM^). Several groups determined the structure of the catalytic domain of human PDE5A1 independently. Sung *et al.* (2003 ▸) chose a construct comprising residues 537–860. In the co-crystal structure with sildenafil, tadalafil and vardenafil, a loop which differs in length and sequence from other PDEs is largely disordered. Zhang *et al.* (2004 ▸) described a similar construct to determine the structure of PDE5A1 in the unbound state, and found that the loop was folded into and blocking access to the active site, further demonstrating the large conformational flexibility of the loop. To facilitate further structural studies, Zhang and coworkers replaced the loop region by the equivalent residues in PDE4B2B, which adopts an ordered helical conformation in the PDE4B structure (Xu *et al.*, 2000 ▸). Unpublished data from our own group show that the flexible loop is prone to proteolytic digest, resulting in batch-to-batch variation of the protein preparation and low reproducibility of ligand co-crystallization. Mass-spectrometric analysis and N-terminal sequencing showed that the protein is proteolytically cleaved at several sites within a short stretch of the protein sequence (Fig. 3 ▸). We also engineered a chimera protein in which the residues of the disordered region were replaced with the equivalent PDE4B2B residues, and found that ligands bound to the chimeric protein with equal affinity compared with the wild-type catalytic domain. The chimera crystallization system was not only highly reproducible, but the new crystal form allowed ligand soaking.

Alternatively, the residues predicted to be disordered can be cut out and the ends joined by short flexible linkers. To obtain the co-crystal structure of capsid protein (CA) N-terminal assembly domain (NTD) with PF-3450074, it was necessary to create a loop-truncation mutant in which a flexible loop is replaced by a single glycine residue (Blair *et al.*, 2010 ▸). The capsid protein of HIV-1 is the primary structural protein of HIV, and is involved in both the assembly of viral particles and the infection of host cells. The N-terminal assembly domain is required for mature capsid assembly and it is possible to interfere with viral uncoating and formation of infective particles in infected cells with small-molecule drugs by targeting the NTD. Although the loop truncated for structural studies is important for viral infection, truncation does not affect compound binding, as validated by affinity measurements, and comparison of the crystal structures with other CA NTD structures with the loop residues present in the protein construct did not show any significant differences.

As these loop-deletion or replacement mutations introduce more extensive changes to the primary sequence of the protein, it is important to verify that they do not affect compound binding biochemically. One should further check for consistency in the structure–activity relationship of the compounds. The artificial nature of the construct should further be kept in mind when setting the structural information into a wider context, such as isoform specificity or selectivity.

### 3.4. Surface mutations   

On their surface, proteins commonly harbour residues with flexible, charged side chains, which are often found in patches. These are suggested to have evolved as a method to prevent undesired protein–protein interactions in the cell (Doye *et al.*, 2004 ▸). Crystal formation, however, is reliant on protein–protein interactions to mediate crystal packing. An early study by McElroy *et al.* (1992 ▸) describes the systematic mutation of nonconserved surface residues and their effect on crystallizability. For human thymidylate synthase, they created a panel of 12 single surface mutants in which charged residues were replaced with neutral amino acids or with amino acids of opposite charge. They found that many of these surface mutants were more crystallizable than the wild-type protein and that a single arginine-to-glutamate mutation produced seven times more crystal hits compared with the wild type. Later, Longenecker *et al.* (2001 ▸) reported an alternative approach in which, for patches of large hydrophilic side chains, the residues were mutated to alanine in order to overcome the resistance of a protein construct to crystallize. For human RhoGDI, they tested 13 mutants with one or several surface residues mutated to alanine, and found new crystal forms for two single and two triple mutants. Derewenda (2004 ▸) discusses a number of other examples where this approach of surface-entropy reduction (SER) have been successfully used. He and others subsequently developed the surface-entropy reduction prediction server *SERp* in order to help and identify possible sites of high surface entropy (Goldschmidt *et al.*, 2007 ▸).

As for construct design in general, it is also recommended to test several combinations of surface mutants in parallel. Sorrell *et al.* (2016 ▸) describe their efforts to generate the first crystal structure of the BIKE kinase domain. BIKE (BMP-2-inducible kinase) is a Ser/Thr kinase of the family of Numb-associated kinases (NAKs), and in an initial attempt the authors prepared over 50 BIKE kinase domain constructs, of which less than 20% showed soluble expression but none could be crystallized. Six different SER mutants were prepared for the construct which showed the highest expression, mutating up to three lysine residues at a time to alanines. Well diffracting crystals were obtained for a double mutant in which the alanine residues are not directly involved in crystal packing but a longer, charged side chain instead of the alanine side chain would have caused repulsion and/or steric clash for one of the mutations.

An example of increasing protein solubility *via* surface mutations is the glucocorticoid receptor (GR). GR receptors belong to the superfamily of nuclear receptors (NRs). They are regulated through hormone binding to the ligand-binding domain (LBD), and the LBD is targeted by drugs such as prednisolone. Bledsoe *et al.* (2002 ▸) reported their approach to increase the soluble expression and purification levels of the GR LBD. Through sequence alignment with other NRs, they identified a single phenylalanine that, when mutated to serine, facilitated soluble expression of the NR in *Escherichia coli* in the presence of ligand. When Schoch *et al.* (2010 ▸) attempted to use this mutant for co-expression with a different ligand, they did obtain soluble protein, but were not successful in generating co-crystals for structure determination. They therefore screened the existing GR LBD structures for clusters of lysine and glutamic acid residues on the surface and distant from the ligand-binding site, and identified a double alanine mutant that showed increased thermal stability in the presence of ligand compared with the wild-type sequence. The new construct could be used to determine the structure of their ligand of interest.

### 3.5. Troubleshooting   

The most obvious problem in crystal structures aimed at determining protein–ligand interactions is when parts of the protein construct block or bind to the active site, as discussed for PDE5 above. Another example is the apo structure of the catalytic domain of PDE2a, where the terminus of a neighbouring chain binds into the active site (Iffland *et al.*, 2005 ▸).

**Figure 4:**
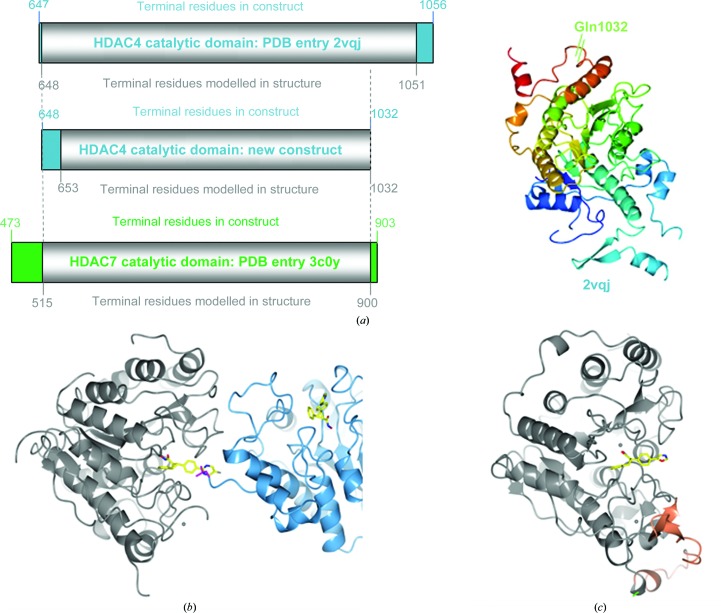
Rounds of construct optimization for the HDAC4 histone deacetylase domain. (*a*) The construct boundaries of the original HDAC4 catalytic domain structure (PDB entry 2vqj, top) and the HDAC7 catalytic domain in PDB entry 3c0y (bottom) are shown. The HDAC7 numbering is aligned with the HDAC4 numbering. The first residue modelled in the original HDAC4 structure corresponds to the first residue modelled in the HDAC7 structure and was used as the starting point for the optimized HDAC4 construct (middle). The C-terminus of the optimized HDAC4 model was chosen based on the last residue visible in the HDAC7 structure. This shortened the new HDAC4 construct by about 20 residues compared with the original HDAC4 structure, and the new C-terminal boundary is highlighted in the cartoon representation of HDAC4 (PDB entry 2vqj). It was speculated that this truncation would help to generate an alternative crystal form with more favourable packing contacts. (*b*) Crystal structure of the new HDAC4 construct with bound ligand (PDB entry 4cbt). The new HDAC4 construct did result in an alternative crystal form. Here, close crystal contacts are observed between ligand (yellow) and Leu728 (magenta) at the terminus of a disordered loop in a neighbouring chain (blue). These contacts were thought to hinder co-crystallization with larger ligands. (*c*) In a second round of construct optimization, Leu728 was mutated to alanine (green) and the mutant readily crystallized in the presence of ligand in yet another crystal form devoid of packing interactions at the ligand-binding site (PDB entry 4cby). In addition, a loop that was disordered in the corresponding wild-type structure could be modelled into the electron density (orange). Figures were created with the *CCP*4*mg* molecular-graphics software (McNicholas *et al.*, 2011 ▸).

In the case of HDAC4, inspection of the first ligand-bound structures of the catalytic domain in the public domain suggested crystal packing to have a distorting effect on the binding mode of the ligand. To overcome this, we designed an alternative protein construct using the boundaries of the successfully crystallized catalytic domain of HDAC7 as a guide (Schuetz *et al.*, 2008 ▸), in particular the first and last residues modelled in the HDAC7 structure (PDB entry 3c0y; Fig. 4 ▸ *a*). Co-crystallization trials with this construct yielded ligand structures that were not biased by the same crystal-packing effects observed for the original construct, but further attempts to co-crystallize other ligands or to repeat the initial trials failed. Inspection of the crystal packing revealed that a leucine residue of a neighbouring chain packs closely against the ligand (Fig. 4 ▸ *b*). Within the protein chain, the leucine was distant from the active site and at the terminus of a disordered loop. Hypothesizing that mutation of the leucine to a less bulky residue would allow a slightly looser packing interaction without affecting protein function, we introduced a leucine-to-alanine mutation, which retained enzyme activity compared with the wild-type protein, as expected. Crystallization conditions were readily identified for the single point mutant in the presence of a variety of different ligands, yielding a novel crystal form devoid of any packing interactions around the active site, and with the formerly disordered loop visible in the electron density (Bürli *et al.*, 2013 ▸).

Another common problem is the need to switch to an alternative crystal form when the original system does not allow crystal soaking. This can be achieved by the identification of alternative crystallization conditions, subtle changes to the construct boundaries as described above or by more drastic changes to the construct. Clifton *et al.* (2015 ▸) describe how an MBP-fusion protein was used to successfully generate a soakable crystal system for Mcl-1. Mcl-1 is a member of the Bcl-2 family of proteins that regulate apoptosis. The sequence N-terminal to the Bcl-2 domain is predicted to be of low structural complexity and to be highly disordered, and structures of Mcl-1 co-crystallized with ligand comprised the Bcl-2 domain only. Involvement of the ligands in packing inter­actions in all but one of these structures suggested crystallization success to be highly ligand-dependent. Clifton and coworkers therefore engineered a MBP-Mcl-1 fusion protein, undergoing multiple rounds of construct optimization. Firstly, they fused a range of different partners including MBP, lysozyme, thioredoxin (Trx) and SUMO N-terminal to the Bcl-2 domain of Mcl-1, but none of the fusion proteins tested yielded crystals in the absence of ligand. To aid crystallization, they further introduced SER mutations on the Mcl-1 protein (K194A, K197A and R201A) in a stretch of ten highly disordered residues. Further, they added linker residues between Mcl-1 and MBP to reduce steric clashes between the two domains: a short GS and a longer GSGGGG linker. The short linker in combination with the SER mutations and addition of the MBP ligand maltose allowed crystallization of the protein in the absence of Mcl-1 ligands. Analysis of the structure showed that the overall fold of Mcl-1 is similar to other Mcl-1 structures in the PDB derived from both NMR as well as X-ray diffraction data. The SER mutations introduced are involved in key packing interactions. Also, the ligand-binding groove is accessible from the solvent channels, and it is possible to generate ligand co-crystal structures *via* soaking with this new Mcl-1 crystal form.

## 4. Protein expression and purification   

There are many different expression systems available that are used for structural studies. The vast majority (75%) of all proteins with structures reported in the PDB were expressed using the prokaryotic expression host *E. coli*, and the ratio is the same if only human proteins are considered. *E. coli* is a relatively easy-to-use expression system and is time- and resource-efficient, and suitable equipment is readily available in standard laboratories. There are a number of different *E. coli* expression strains with enhanced functionality available to help overcome issues such as codon bias, proteolysis, toxicity of the expressed gene or disulfide-bridge formation. Overviews of protein expression in *E. coli* for structural studies are given in Gräslund *et al.* (2011 ▸) and by Papaneophytou & Kontopidis (2014 ▸).

The *E. coli* expression system has limitations, as it is restricted in terms of the maximal size of the protein expressed, is less suitable for the expression of proteins containing membrane-associated domains and is quite limited if protein phosphorylation or glycosylation is required. Table 2 ▸ summarizes the most common alternative expression systems and compares their advantages and disadvantages. Essentially, for many proteins, the choice of expression system from which to obtain material suitable for crystallization studies is frequently driven by pragmatism, and it is beyond the scope of this article to discuss it in detail.

If soluble protein can be expressed only in low yields, the expression level may be increased by addition of ligand during protein expression, and nuclear receptors are a prominent example where expression levels are highly ligand-dependent (Hassell *et al.*, 2007 ▸; Bledsoe *et al.*, 2002 ▸). Stability and cellular uptake of the compound during expression can be other issues (Cowan-Jacob *et al.*, 2007 ▸), and fairly large amounts of the ligand are required for co-expression. It is therefore worth considering alternative ligands to that of interest, preferably with a low(er) binding affinity, in order to be able to dilute the ligand out at a later stage and replace it with the ligand of interest. Ligand exchange can be facilitated by incubating the purified complex with the ligand of interest directly, or by dialysing the purified complex first against a buffer without compound. In the case of nonphosphorylating glyceraldehyde-3-phosphate dehydrogenase (GAPN) from *Thermoproteus tenax*, removing NADPH carried through during protein expression and purification from the active site of GAPN required heat treatment of the protein. During this process, NADPH could be successfully replaced with the co-substrate NAD (PDB entry 1uxt; Lorentzen *et al.*, 2004 ▸). In general, heat treatment can help to remove impurities of misfolded protein and increase the overall homogeneity (Hassell *et al.*, 2007 ▸).

If the protein can be solubly expressed but deteriorates during purification, the addition of a ligand during lysis or to the purification buffer at a later stage can help to stabilize the protein. As mentioned before, the amounts of ligand required and the associated costs can be significant. Fluorescence- or light-scanning-based methods that determine the thermal stability of a protein are often used to optimize protein buffer conditions in terms of pH or salt composition (Pantoliano *et al.*, 2001 ▸; Ericsson *et al.*, 2006 ▸), and can also be used to screen and rank alternative ligands. In case no other ligand or inhibitor is known for the protein target of interest, these methods can also be used to screen generic ligands and additives that might help to stabilize the protein. Vedadi *et al.* (2006 ▸) reported that screening of a panel of physiologically relevant small molecules directly contributed to a crystal structure in a number of cases, and suggested screening the (patent) literature for suitable small molecules if the activity of the protein is known. In addition, thermal stability assays can be used to identify suitable protein buffer conditions, as ligand binding may be dependent on the buffer composition or pH (Müller *et al.*, 2011 ▸).

After successfully generating a homogenous preparation of pure protein, it should be confirmed that the purified protein fragment is able to bind to the ligand of interest. If working on the development of a protein inhibitor, one might have access to a functional assay in which the protein fragment can be tested, or a binding assay such as isothermal titration calorimetry (ITC) can be used.

## 5. Soaking *versus* co-crystallization   

Ligand co-crystallization typically requires more resources compared with soaking, both in terms of time and material. Miniaturization and automation of the crystallization setup help to minimize protein consumption and to achieve reasonable throughput. In addition, crystal seeding techniques are a powerful tool to speed up the optimization process and to obtain good-quality crystals more consistently.

Once crystallization conditions have been optimized, it is quite common that several good-quality crystals appear in the crystallization drops. Using all of them for separate soaking experiments obviously reduces the number of crystallization experiments required and helps to maximize the number of co-crystal structures per protein preparation compared with co-crystallization. The primary caveat of soaking is the need for a crystal form with an accessible ligand-binding site. On the ligand side, reasonable ligand solubility is required, in particular for low-affinity binders.

If co-crystal structures for only a small number of compounds are to be determined in a new protein system, and if sufficient purified protein is available, it is recommended to screen for apo as well as protein–ligand complex crystallization conditions in parallel. This is not only useful to increase the chances of complex crystals, but in case issues arise with the crystal system an alternative system is already at hand.

### 5.1. Soaking   

A prerequisite for soaking is the existence of a soakable crystal form. Inspection of a crystal structure can indicate whether soaking should be possible: close crystal-packing contacts around the ligand-binding site (PDE2A, as mentioned above) or around regions expected to undergo conformational change upon ligand binding might be problematic. When checking for potential crystal-packing issues, it is recommended to not only look at the atomic model but also at the electron-density maps. Flexible regions or affinity tags might not have been modelled, and features in the electron-density maps might reveal whether these are close to and possibly interfering with the ligand-binding site.

The protein does not necessarily need to have been crystallized in the ligand-free form to be suitable for soaking. Co-crystallized ligands can often be displaced, even by ligands with equal affinity. In the case of apoptosis signal regulating kinase 1 (Ask1), the co-crystallized inhibitor was replaced by an equally potent compound (IC_50_ for both in the sub-micromolar range) by soaking at 10 m*M* for a couple of days (PDB entries 4bib and 4bic; Singh *et al.*, 2013 ▸). Indeed, for ligands which induce large conformational changes that are not tolerated by crystal packing in the apo form, co-crystals of a similar ligand would seem to be a better starting point for soaking trials. For allosteric modulators, it might be necessary to have a ligand present in the active site, either to block the site to avoid false-positive hits or to induce a particular conformation required for binding. AGC kinases have a small phosphate-binding site in the N-lobe, which is believed to play a key regulatory role. This binding pocket is disrupted in the crystal structures of AGC kinases in the inactive conformation but is ordered in the active conformation. For the AGC kinase PDK1, small molecules that bind to this phosphate-binding pocket were successfully introduced by soaking into crystals of ATP-bound PDK1, *i.e.* the protein in its active conformation (PDB entry 3hrf; Hindie *et al.*, 2009 ▸).

#### 5.1.1. Experimental setup   

The simplest way to soak a compound into a crystal is by adding either a concentrated stock solution or pure compound directly to the crystals in the crystallization drop. This reduces crystal manipulation, especially for fragile crystals, and allows automation of the process (Krojer *et al.*, 2017 ▸). The main disadvantage is that only one ligand can be explored per drop, potentially wasting many crystals if multiple crystals are present. When the ligand is added, especially in the solid form, it is recommended to first remove any skin that might have formed on the crystallization drop, both to allow diffusion of the compound and to not hinder crystal harvesting later. In the case where cryoprotection is applied after crystal soaking, ligand should also be present in the cryo-buffer to avoid soaking the ligand back out of the crystals. Depending on the binding kinetics, this can happen on a very short timescale.

Alternatively, the crystals can be harvested into a stabilizing solution before soaking. This solution can either be the crystallization well solution, possibly supplemented with cryoprotectant, or have a more complicated composition, as binding of ligand to the protein can change protein solubility. This is sometimes revealed by disappearing protein crystals upon ligand addition. Increasing the precipitant concentration can counteract this increase in protein solubility and help to maintain diffraction of the crystal after soaking, and in some cases, effectively through crystal dehydration, improve resolution (Russo Krauss *et al.*, 2012 ▸). When salt is used as the major precipitant in the crystallization, high concentrations can decrease solubility, especially for hydrophobic ligands, and also cause issues for cryoprotection. It has been shown that addition of polyethylene glycol (PEG) to an aqueous solution as a co-solvent can help to increase compound solubility (Rytting *et al.*, 2005 ▸). Replacing salt with PEG in order to increase ligand solubility is often possible without damaging the crystals. Also, the pH of the stabilizing solution can be shifted into the range required for ligand binding. If a protein was co-crystallized in the presence of another ligand, the stabilizing solution can be used to soak this ligand out, possibly in several steps, by transferring the crystal into fresh drops over hours or days before soaking with an alternative ligand.

The ligand concentration required for soaking depends on the affinity of the ligand and, together with the soaking time, needs to be optimized. The aim is to achieve that in excess of 90% of protein (P) in the sample is bound to ligand (L). This will depend on the ligand concentration [L] and the dissociation constant of the PL complex *K*_d_, 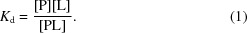



The ratio of bound to total protein can therefore also be expressed as
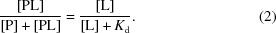



**Figure 5:**
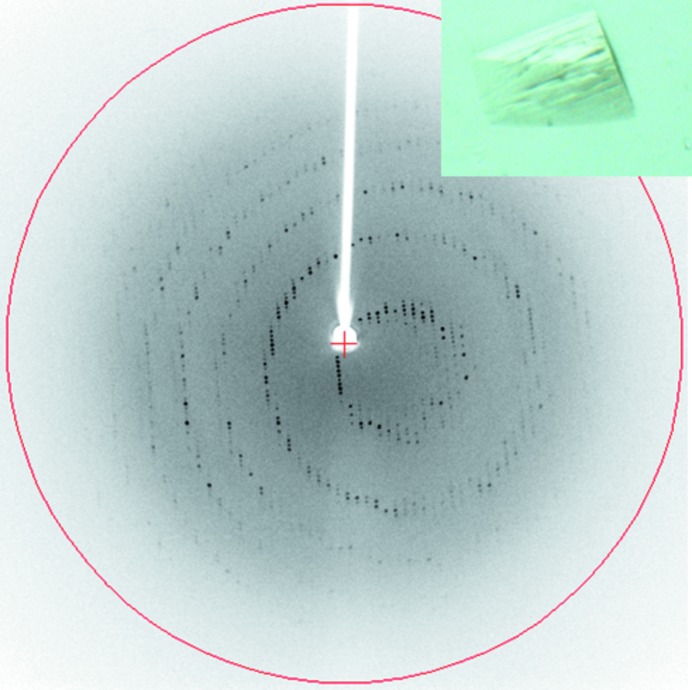
Diffraction image for a crystal apparently damaged during soaking. For the example shown, a data set could be collected and the structure solved without significant loss in resolution or map quality compared with nonsoaked apo crystals.

For >90% of protein to be bound in the complex, the required free ligand concentration is ten times the *K*_d_ or greater. In practice, ten times the total ligand concentration is a good guide for soaking experiments. Yet, often, the binding affinity is not known. In these cases, we would aim for 20–50 m*M* for fragment-like compounds and 0.1–1 m*M* for higher molecular-weight ligands, their solubility permitting. Rapid visual deterioration of the crystals would suggest that the concentration chosen was too high, as would heavy precipitation of the ligand in the stabilization buffer. Crystals harvested at different time points (<1 h, several hours, overnight) can be checked both for diffraction as well as ligand occupancy. Also, a slow, stepwise increase in ligand concentration can help to maintain diffraction. If crystals show signs of deformation (especially plate-like or needle-like crystals) or appear cracked, it is still worth checking the diffraction before discarding the experiment (Fig. 5 ▸). If soaking optimization did not help to reduce crystal decay, data collection at modern synchrotrons and using a single-photon-counting pixel detector can further help to obtain processable data and, more importantly, interpretable electron-density maps. If the diffraction is poor despite optimizing the ligand concentration and stabilizing solution, cross-linking with glutaraldehyde prior to soaking might help to stabilize the crystals (Lusty, 1999 ▸). In essence, protein crystals are equilibrated through the gas phase against a solution containing glutaraldehyde. The glutaraldehyde reacts with and forms cross-links between lysine side chains on the protein surface, ideally tightening the crystal contacts and making the crystal more resilient to conformational changes during soaking. In terms of experimental setup, we generally add 5 µl of 10–25% glutaraldehyde to a microbridge or sitting-drop well and add 50 µl of crystallization or stabilization solution to the reservoir. Crystals are placed on a cover slip, either in the crystallization or a stabilizing solution, suspended over the well and equilibrated from between 5 and 10 min up to several hours before harvesting for the soaking experiment.

#### 5.1.2. Ligand preparation   

The ligand can be added to the protein crystals as a solid. An obvious disadvantage of this method is that it is very difficult, if not impossible, to achieve a defined ligand concentration, making it hard to optimize ligand concentration for sensitive crystals. We prefer to add compound dissolved in an organic solvent at concentrations of greater than 50 m*M*, solubility permitting. The presence of a solvent can help to increase ligand solubility in the soaking solution. DMSO is the solvent that is most commonly used, as it is not particularly volatile, is able to dissolve both polar as well as apolar compounds and is suitable for compound storage. Most protein crystals can stand the presence of 1–2% DMSO, and some even up to 10–20%. DMSO can be supplemented by other solvents to increase ligand solubility further or if the crystals do not tolerate high DMSO concentrations. Ciccone *et al.* (2015 ▸) describe dioxane and 2,3-butane­diol as co-solvents, and suggest their use in combination with other cryoprotecting components. Öster *et al.* (2015 ▸) reviewed different experimental approaches used to overcome typical issues observed for ligand soaking, in particular in the context of fragment-based drug discovery, where a robust crystal soaking system allowing high throughput of low-affinity ligands is essential. They analysed the properties of ligands that were successfully solved *versus* those which did not yield co-crystal structures. The major predicting factor was potency, as one would have assumed, but the crystallization success was also weakly correlated with the calculated octanol–water partition coefficient, *c*log*P*, in favour of more lipophilic compounds. These results suggest that screening for solubilizing solvents is worthwhile. However, for very in­sol­uble compounds, replacement with a more soluble analogue might be the only way to a co-crystal structure.

Occasionally, DMSO is found coordinated to the ligand-binding site and, especially when screening for fragment-like low-affinity ligands, replacing DMSO with an alternative solvent should be considered (PDB entries 4ior and 4ioq; Lolli & Battistutta, 2013 ▸).

In case the crystals do not tolerate any organic solvent, but the solid compound is not easily dispensed or is poorly soluble in water, the compound can first be dissolved at a high concentration in a volatile solvent (methanol or ethanol). To prepare the soaking solution, the required volume of this stock is pipetted out, the solvent is allowed to evaporate and the dried compound is resuspended in the stabilizing buffer.

If volatile solvents are used to help with compound solubility or are present in the crystallization conditions, evaporation from the soaking drop during harvesting can cause the crystals to spin in the drop and make harvesting very difficult. To reduce evaporation, and if toxicity allows, a tissue soaked in a mixture of water and the solvent can be placed close to the plate or cover slip when harvesting the crystals.

#### 5.1.3. Sanity check   

During soaking, ligands bind to the protein after the crystal lattice has formed. Extra care needs to be taken when interpreting the structural data to ensure the binding mode is genuine and is not a result of crystal-packing artefacts. In the example of PDE10A, the protein crystallizes with two chains per asymmetric unit, and the catalytic site of one of them is in close contact with a neighbouring chain. Upon soaking of these PDE10A crystals, it is often observed that ligand is only bound to the chain not affected by crystal packing (PDB entry 3snl; Malamas *et al.*, 2011 ▸). However, in some cases the ligand binds to both sites with differing binding modes (PDB entries 2wey and 4bbx; Andersen *et al.*, 2009 ▸; Bartolomé-Nebreda *et al.*, 2014 ▸), for which the most likely explanation is steric restriction. One is rarely as lucky as in the case of PDE10A, where the ligand-binding mode near a packing interface can be checked against an unaffected ligand-binding site within the same crystal. In all other cases it is advised to test the binding hypothesis carefully. This can be performed by comparison with similar ligands bound to the same or homologous proteins, or in case where no further crystal structures are available, by checking for agreement with the (ligand) structure–activity relationship (SAR). If in doubt, the complex structure should be validated through co-crystallization, but even then crystal packing can distort the binding mode, as in the case of HDAC4 discussed above.

### 5.2. Co-crystallization   

For co-crystallization, the ligand can be added to the protein during crystallization setup, and Gelin *et al.* (2015 ▸) even suggest ‘dry’ co-crystallization by pre-coating crystallization wells with ligand. More commonly, the protein is pre-incubated with the ligand. Initial crystallization screens are performed in the same way as any protein-crystallization experiment (Ng *et al.*, 2016 ▸), avoiding conditions that are known to disrupt ligand binding (Müller *et al.*, 2011 ▸). If available, the drops can be cross-seeded using crystals of either the apo-protein or another ligand complex.

#### 5.2.1. Experimental setup   

The protein concentration during pre-incubation with compound can either be the concentration used in the crystallization experiment or more dilute. This mainly depends on the solubility of the compound, as a higher protein-to-compound ratio can be achieved for the diluted protein. It can also become necessary if the compound stock solution is at a low concentration or is in a solvent that is not well tolerated by the protein. If possible, we prefer to incubate the concentrated protein with the compound, especially when screening several ligands in parallel or in cases where the compound interferes with the membrane of the protein concentrator. The compound concentration during pre-incubation depends on the binding affinity, and as a rule of thumb should be at least three times the *K*_d_. We usually leave the complex to form at 4°C overnight or at least for 1 h at room temperature. Hassell *et al.* (2007 ▸) report that temperature can also have an effect on complex formation and that heat-treatment of the complex before crystallization setup can help to obtain a more homogeneous complex, as mentioned above.

To remove any precipitated compound or protein, the sample can be centrifuged or filtered before setting up the crystallization screens. If crystallization conditions for the protein are known, we would set up factorial and custom screens in parallel and in combination with microseeding.

Seeding with existing microseed stocks into matrices of unrelated crystallization conditions has been termed microseed matrix screening (MMS), and its usefulness as well as its automation have been described and reviewed in detail by D’Arcy *et al.* (2014 ▸). In essence, a seed stock is prepared from initial crystallization hits and added to the crystallization drops (a typical ratio of seed to protein to well solution is 0.3:0.7:1) *via* automated crystallization setup using pipetting robots such as the Mosquito (TTP Labtech, Melbourn, England) or Oryx series (Douglas Instruments, Berkshire, England). With the addition of 15%(*v*/*v*) seed stock, seed-stock buffer components are transferred in significant amounts alongside any microcrystals and the success of MMS might also be attributed in part to this additive-screening component of the experiment.

Rumpf *et al.* (2015 ▸) describe how they used MMS to improve crystal quality for human sirtuin isotype Sirt3 and changed to more favourable crystallization conditions for soaking at the same time. They further compared the results obtained for one factorial 96-well screen with and without MMS: only two crystallization conditions were identified without seeding, but in excess of 20 with seeding. It is worth mentioning that they also tested MMS at two different temperatures (4 and 20°C), and there is only partial overlap between the successful crystallization conditions at the different temperatures.

The choice and number of conditions tested depends on whether there is prior knowledge of successful crystallization, the dependence of the protein–ligand complex on the buffer composition, the amount of protein and ligand available, and ultimately the preference of the experimenter for particular factorial sparse-matrix screens. If it was known that previous ligands could be co-crystallized in PEG conditions, initial attempts could include a PEG screen such as the PACT screen (Newman *et al.*, 2005 ▸). Using PEG and low-salt conditions can also be beneficial for co-crystallizing lipophilic compounds.

Analysis by Ng *et al.* (2016 ▸) suggests that setting up screens with varying drop ratios (2:1, 1:1 and 1:2 protein:well solution) at two different temperatures increases the likelihood of the identification of conditions, especially for proteins that are hard to crystallize.

#### 5.2.2. Troubleshooting   

A typical issue with co-crystallization is reduced ligand occupancy. As for the soaking approach, the compound should be present in the cryo­protectant, but there can also be crystal-to-crystal variation, even within the same crystallization drop. Even for low-nanomolar affinity compounds, we found an occupancy ranging from well below 50% to near-full occupancy between crystals (unpublished results). If the crystals consistently show low ligand occupancy, an additional soaking step could be tested before resorting to further crystallization trials (unpublished results). On the other hand, if the protein sample contains a large fraction of unbound protein, this ‘impurity’ can have a negative effect on crystallization reproducibility, and the addition of further ligand can help to shift the ratio towards the complexed protein (Mann *et al.*, 2016 ▸).

If ligand present during protein expression is bound to the protein, it might not be sufficient to pre-incubate the protein with the compound of interest, but addition to the purification buffer or a dialysis step after purification might be required to remove the endogenous ligand. In the case of the ligand-binding domain (LBD) of the GluN1 receptor, glycine is the native receptor agonist and binds with high affinity, inducing a large conformational change compared with the unbound or antagonist-bound state. To obtain the structure of the GluN1a LBD in the complex with an antagonist, Kvist *et al.* (2013 ▸) purified the LBD in the presence of glycine and then dialyzed against a glycine-free buffer before incubating with the antagonist at saturation. To obtain the structure of the unbound state, it was necessary to add the lower affinity ligand l-serine to all chromatography buffers during purification and to add an extensive dialysis step (volume change of 10^15^ over 3–4 d) prior to crystallization setup (Yao *et al.*, 2013 ▸).

If co-crystallization has worked for one ligand, by no means is it guaranteed that it will work for others, and warning signs can occasionally be found through careful inspection of the known structure. In case of Fyn kinase, the Protein Data Bank lists one crystal structure for its kinase domain with stauro­sporine bound to the ATP-binding site (PDB entry 2dq7; Kinoshita *et al.*, 2006 ▸). Closer inspection of electron-density maps indicates the presence of another staurosporine-binding site near Trp4 and Trp30, which has not been included in the model but which contributes strongly to crystal-packing contacts. This suggests that staurosporine is required for crystal formation, and replacing staurosporine with another ATP-site binder under the same crystallization conditions might not yield crystals. In a first attempt, it would be recommended to screen for wider crystallization conditions.

## 6. Conclusions   

Crystallization of protein–ligand complexes requires careful consideration of a variety of parameters from protein construct design, choice of expression system and optimization of purification conditions to fine-tuning of (co-)crystallization and soaking. The review of recent analyses of large-scale protein structure-determination efforts has identified a number of factors that are important for a systematic approach to establishing a robust crystal system. Prioritization of the different aspects of protein construct design is thought to be key for developing a crystal system early to maximize the impact of structural information in a project, rather than merely describing structural aspects in hindsight. The examples discussed should make the variables and issues in protein crystal complex formation more tangible, but also serve as an inspiration for anyone stuck in the process. Yet, it should not be forgotten that protein crystallography does involve trial and error as a key aspect and at each stage. Therefore, as many (well chosen) conditions as possible should be tested, but no more than the laboratory is set up to deal with and the experimenter is comfortable handling at any time.

## Figures and Tables

**Table 1:** Experimental considerations for different entry points to the protein–ligand complex structure-determination cycle illustrated in Fig. 1 ▸

Starting point/assessment result	Checks	Experimental considerations
*Previous structure *via* soaking*		
Good-quality data, structure explains SAR	Do new compounds differ from previous (affinity, MW, solubility)?	Use same construct. If soaking fails, try optimizing soaking time and ligand concentration. If not successful, try co-crystallization.
Poor occupancy of ligands	Check affinity/solubility of ligands	Increase ligand concentration and/or soaking time
Binding mode cannot explain SAR	Check structure: packing issues around ligand-binding site?	Try generating a new crystal form(i) change crystallization conditions (ii) co-crystallize ligand (iii) change construct
	Check construct used: mutations/modifications that might impair ligand binding?	Test alternative constructs

*Previous structure *via* co-crystallization*		
Good-quality data, structure explains SAR	Do new compounds differ from previous (affinity, MW, solubility)?	Use same construct. Co-crystallization might be ligand-dependent and a wider crystallization screen might be necessary.
Poor occupancy of ligands	Check affinity/solubility of previous ligands	Pre-incubate protein at higher compound excess. Reduce protein concentration during incubation for compounds with low solubility. Co-crystallize (with lower affinity compound) and back-soak.
Binding mode cannot explain SAR	Close crystal-packing contacts near ligand-binding site?	Try generating a new crystal form by changing crystallization conditions
	Mutations/modifications that might impair ligand binding?	Test alternative constructs

*No structure available*		
	Full-length structure or isolated domain of target protein needed?	Consider preparing full-length protein alongside domain fragments as it can be used as a reference in biochemical assays, and its limited proteolytic digest can assist in the choice of suitable boundaries for the protein fragments
	Homologue structure available? Do proteins align well at termini of homologue construct? Secondary-structure prediction: low complexity/secondary-structure elements at domain boundary?	Chose boundaries similar to homologue in case of good alignment, but avoid cutting into predicted α-helices and β-strands or including long stretches of low structural complexity. Follow guidelines given in §3.1 on terminal residues.
	PTM to be considered?	Include mutations that mimic/prevent PTM

**Table 2:** Comparison of expression systems

Expression system	Advantages	Disadvantages
*E. coli*	Simple, cheap, easily available to most laboratories, many specialized strains and plasmid expression vectors available, many with strong inducible promoters. Quick and easy to scale-up; good for multi-construct screening. Potential for inclusion-body/refolding route to protein generation.	Lack of post-translational modification (glycosylation, phosphorylation *etc.*). Rarely the best choice for secreted or membrane proteins. Not always suitable for soluble expression.
Yeast	Media cheap, scale-up easy and expression levels often very high. Good for some post-translational modifications. Can be good for secreted and membrane proteins	*Pichia* needs initial effort to identify high-expressing clones: not great for multiple constructs. Cell breakage difficult if intracellular.
Insect cells/baculovirus	Generally good expression levels. Relatively easy to propagate cells. Good for most post-translational modifications. Good for secreted and membrane proteins or where there is a requirement for co-expression of multiple subunits (can use multiple viruses).	Media expensive; virus generation is quite time-consuming. Lytic system reduces opportunities for stable expression: inducible systems limited. Requires sterile environment for propagation.
Mammalian	Good for screening expression (as transients). Can obtain stable expression (integrated). Best for authentic post-translational modification. Inducible systems available. Good for secreted and membrane proteins.	Media often very expensive; transient scale-up requires expensive transfection reagents and large quantities of plasmid DNA. Complex glycosylation can interfere with crystallization. Requires sterile environment for propagation and CO_2_ atmosphere in many cases.
